# Antifouling Activity of Halogenated Compounds Derived from the Red Alga *Sphaerococcus coronopifolius*: Potential for the Development of Environmentally Friendly Solutions

**DOI:** 10.3390/md20010032

**Published:** 2021-12-28

**Authors:** Maxence Quémener, Stefanos Kikionis, Marilyne Fauchon, Yannick Toueix, Fanny Aulanier, Antonios M. Makris, Vassilios Roussis, Efstathia Ioannou, Claire Hellio

**Affiliations:** 1 Laboratoire des Sciences de l’Environnement Marin (LEMAR), Université de Brest, CNRS, IRD, Ifremer, F-29280 Plouzané, France; quemener.max@gmail.com (M.Q.); fauchon@univ-brest.fr (M.F.); yannick.toueix@univ-brest.fr (Y.T.); fanny.aulanier@gmail.com (F.A.); 2 Section of Pharmacognosy and Chemistry of Natural Products, Department of Pharmacy, National and Kapodistrian University of Athens, Panepistimiopolis Zografou, 15771 Athens, Greece; skikionis@pharm.uoa.gr (S.K.); roussis@pharm.uoa.gr (V.R.); 3 Institute of Applied Biosciences, Centre for Research & Technology, Hellas (CERTH), 570 01 Thessaloniki, Greece; makris@certh.gr

**Keywords:** antifouling, adhesion, marine natural products, halogenated diterpenes, *Sphaerococcus coronopifolius*

## Abstract

Nowadays, biofouling is responsible for enormous economic losses in the maritime sector, and its treatment with conventional antifouling paints is causing significant problems to the environment. Biomimetism and green chemistry approaches are very promising research strategies for the discovery of new antifouling compounds. This study focused on the red alga *Sphaerococcus coronopifolius,* which is known as a producer of bioactive secondary metabolites. Fifteen compounds, including bromosphaerol (**1**), were tested against key marine biofoulers (five marine bacteria and three microalgae) and two enzymes associated with the adhesion process in macroalgae and invertebrates. Each metabolite presented antifouling activity against at least one organism/enzyme. This investigation also revealed that two compounds, sphaerococcinol A (**4**) and 14*R*-hydroxy-13,14-dihydro-sphaerococcinol A (**5**), were the most potent compounds without toxicity towards oyster larvae used as non-target organisms. These compounds are of high potential as they are active towards key biofoulers and could be produced by a cultivable alga, a fact that is important from the green chemistry point of view.

## 1. Introduction

In the marine environment, all untreated man-made submerged surfaces are colonized by fouling organisms, such as bacteria, algae and invertebrates, including barnacles and mussels [1,2]. This phenomenon is defined as biofouling [3,4]. It is a natural process by which species whose life stage requires a fixed mode will adhere to surfaces in either a reversible or irreversible way. Biofouling is the focus of many studies, particularly to understand the attachment processes of invertebrate larvae or algal spores/zygotes. Actually, in the context of increasing mariculture’s activities, understanding these processes will allow for the improvement in sustainability of the aquaculture sector [5,6]. In the marine environment, there is very strong ecological pressure and competition during the adhesion phases, as the number of organisms that need to adhere is much higher than the available surfaces. Thus, many marine organisms have developed the mechanisms to adhere not only to natural substrates but also to artificial surfaces. In the latter case, unwanted colonization leads to deterioration and corrosion of these surfaces, resulting in significant economic losses [1,7]. Thus, biofouling has been the subject of continuous multidisciplinary research, particularly in maritime fields related to transportation (protection of hulls), marine energy (protection of structures), aquaculture (protection of nets and cages), infrastructure (jetties, dinghies, harbors) and water intakes and cooling circuits of thermal power plants [8,9]. 

The colonization of ship hulls has been extensively studied. Thus, marine biofoulers are classified in three categories, based on their impact on the increase of frictional drag (FD) of man-made immersed surfaces: (i) microfoulers which are responsible for 1–2% increase in FD (bacterial, fungal and microalgal biofilms); (ii) soft macrofoulers which are responsible for up to 10% in FD (macroalgae); and (iii) hard macrofoulers leading to up to 40% increase in FD (barnacles, etc) [1]. 

To prevent the settlement and growth of biofoulers and/or to reduce their impact on man-made surfaces, antifouling coatings have been extensively used. These paint matrices are formulated with heavy metals (copper and zinc mainly) and co-biocides. In the 1960s, the chemical industry developed efficient antifouling paints based on tributyltin (TBT) and triphenyltin (TPT). Unfortunately, these two chemicals appeared highly toxic for aquatic organisms and have been proven to bioaccumulate through the food chain and be persistent in the environment and as such were banned in 2008 [10]. Since then, TBT-based paints were replaced by new formulations containing copper or biocides, such as diuron and irgarol [10,11,12,13]. To increase their activity, inorganic zinc is often added to the formulations [14]. However, even if these new formulations were initially claimed to be environmentally friendly, there is now evidence of a widespread use of these compounds leading to significantly high concentrations measured in marinas and harbors [15,16]. 

Nowadays, it is an imminent necessity to develop non-toxic antifouling formulations, eliminating or minimizing the use of heavy metals and organic biocides. A fascinating concept of scientific inspiration is the combination of biomimetic and green chemistry approaches. Indeed, many marine organisms that live attached and lack physical defenses have evolved chemical defenses that allow them to protect their surfaces from epibionts. Thus, secondary metabolites with significant levels of anti-epibiosis activity have been isolated and could be incorporated in antifouling paints [17,18,19,20]. These molecules have the advantage of exhibiting targeted activities and acting at very low concentrations, and thus their use in paint compositions is promising from this point of view [2,21,22,23,24,25]. The ideal solution would be to use natural products to prevent the settlement of fouling organisms rather than inhibiting their growth [2,21,22,23,24,25]. Settlement inhibitors are not considered as biocides, and thus regulations for their market authorization are less restrictive, allowing for faster development and deployment. Developing the use of natural products is also an important aspect of green chemistry, allowing the reduction of the environmental and health impacts of chemical production. It is essential to strive to increase the use of renewable raw materials, to design antifouling paints based on safe and environmentally friendly substances, allowing for the reduction of by-products and degradation products to avoid pollution.

In search of new natural compounds, the marine environment appears as a prolific source for novel bioactive natural products. Actually, marine macro- and microorganisms produce a wide and diverse range of secondary metabolites with promising bioactivities [26,27,28]. Among them, algae are well-known for their capacity to produce molecules with anticancer, antifouling, antibacterial or anti-inflammatory activity [29,30,31].

We have been working for many years on the chemistry and chemical ecology of macroalgae, and we are particularly interested in the defense molecules against epibiosis. Among these biotechnologically interesting algae, the cosmopolitan red alga *Sphaerococcus coronopifolius* is a prolific source of secondary metabolites, mainly diterpenoids. Among these diterpenes, bromosphaerol (**1**) has been proven as a promising antifoulant, surpassing the strictest standards [32]. In these studies, bioassays conducted on the cirriped crustacean *Amphibalanus amphitrite* showed that bromosphaerol (**1**) exerted significant anti-settlement activity with an EC_50_ value of 0.23 mg/L, combined with extremely low toxicity (LC_50_ > 100 mg/L), with an astonishing therapeutic ratio (TR_C_ = LC_50_/EC_50_) of 434.78.

The aim of this study was to further investigate the antifouling potential of bromosphaerol (**1**) and other major metabolites isolated from the red alga *S. coronopifolius* against prominent marine biofoulers (five marine bacteria and three microalgae) and two enzymes associated with adhesion process in macroalgae and invertebrates.

## 2. Results and Discussion

In order to broaden the knowledge on the mode of action and ecological roles of molecules isolated from *S. coronopifolius*, we studied the epibiosis defense activities of 15 secondary metabolites isolated from this alga (**1**–**15**) on biofouling model organisms. This fundamental research can have important applications in the marine biotechnology sector with the discovery of new molecules of interest in the field of antifouling paints’ formulation.

### 2.1. Isolation and Identification of Metabolites from Sphaerococcus coronopifolius

**Figure 1:**
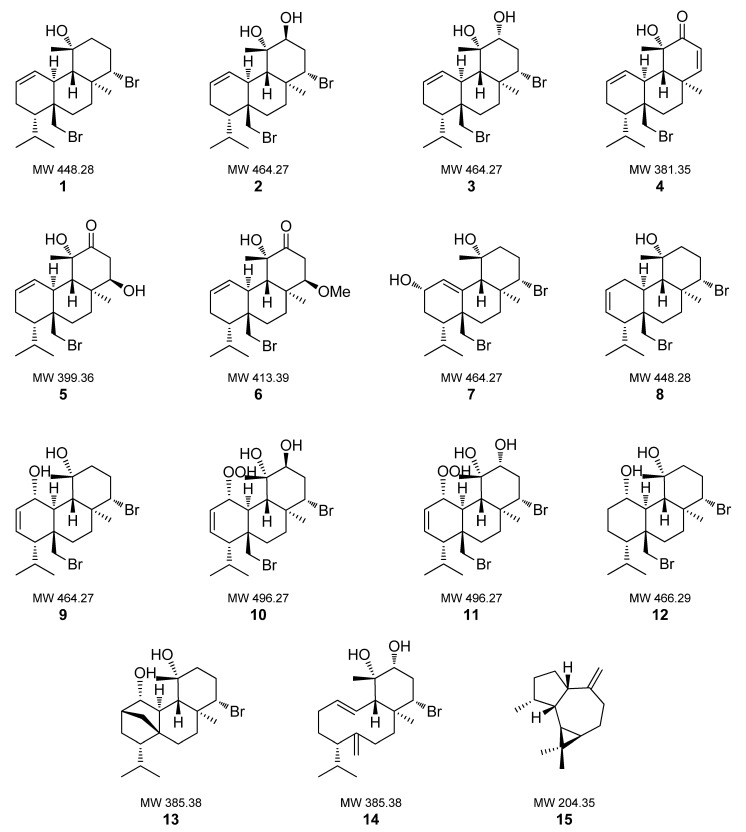
Chemical structures of compounds **1**–**15** isolated from *Sphaerococcus coronopifolius*.

A series of chromatographic separations of the CH_2_Cl_2_ / MeOH extract of the red alga *S. coronopifolius*, collected in Agios Giannis bay off Parga in Greece, resulted in the isolation of 14 diterpenes (**1**–**14**) and one sesquiterpene (**15**) as the major constituents. By comparison of their spectroscopic and physical characteristics with those reported in the literature, the isolated metabolites were identified as bromosphaerol (**1**) [33,34], 12*S*-hydroxy-bromosphaerol (**2**) [35,36], 12*R*-hydroxy-bromosphaerol (**3**) [36,37], sphaerococcinol A (**4**) [38,39], 14*R*-hydroxy-13,14-dihydro-sphaerococcinol A (**5**) [36], 8-methoxy-dihydro-sphaerococcenol (**6**) [40], 2*S*-hydroxy-isobromosphaerol (**7**) [41], bromosphaerol B (**8**) [36], bromosphaerodiol (**9**) [42], 1*S*-hydroperoxy-12*S*-hydroxy-bromosphaerodiol B (**10**) [36], 1*S*-hydroperoxy-12*R*-hydroxy-bromosphaerodiol B (**11**) [36], 1*S*-hydroxy-1,2-dihydro-bromosphaerol (**12**) [41,43], bromotetrasphaerol (**13**) [37], 12*R*-hydroxy-bromocorodienol (**14**) [44] and *allo*-aromadendrene (**15**) [45] (Figure 1).

### 2.2. Evaluation of Antifouling Activity

Antifouling tests were conducted on bacteria, microalgae and enzymes related to macroalgae and invertebrates’ adhesion [46]. It is essential to work on the three biological compartments (microorganisms, macroalgae and invertebrates) because despite established installation sequences (in chronological order, microorganisms, macroalgae and then invertebrates), it has been previously highlighted that the inhibition of a single type of organism does not prevent the colonization of surfaces by other biofoulers. It is therefore essential to have an integrated approach, in terms of both complementary bioassays and types of molecules. Indeed, marine organisms use combinations of antifouling defenses that act synergistically, making it important to evaluate the activity of molecules as many as possible isolated from the same organism [47].

The choice of biotests (target organisms and types of bioassays) is an essential step in bioprospecting projects and has a crucial impact on the analysis of results [48]. Thus, it is important to carefully think about the desired mode of action and, if necessary, to work on the development of innovative methods to evaluate the activity. Within the framework of the development of new antifouling solutions that are eco-friendly and in order to avoid secondary effects on non-target organisms as much as possible, the biological activity sought is not a biocidal activity (as it was usually done) but an adhesion-inhibitory or deterrent activity [2,21,22,23,24,25]. This approach is also interesting in the context of marketing authorization applications. Indeed, these are very long, strict and complex processes in the case of biocides (same legislation as for pesticides) but much simpler in the case of adhesion inhibitors. Thus, for this work, we focused on the evaluation of modes of action involving adhesion inhibition. We also favored an ethical approach by minimizing tests on living organisms. To do so, biochemical assays targeting enzymes involved in the synthesis of adhesives by algae and invertebrates were chosen, i.e., bromoperoxidase and phenoloxidase, respectively.

#### 2.2.1. Inhibition of Adhesion and Growth of Marine Bacteria and Microalgae

**Figure 2:**
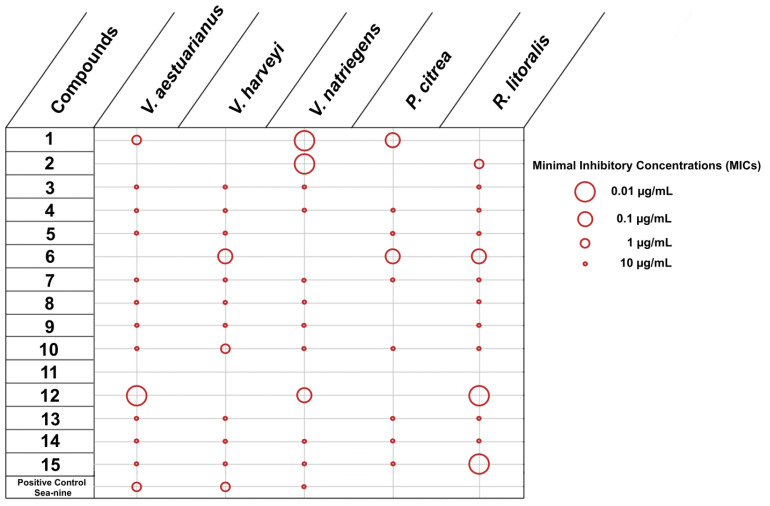
Inhibition of adhesion of bacteria by compounds **1**–**15** (MICs in µg/mL).

**Figure 3:**
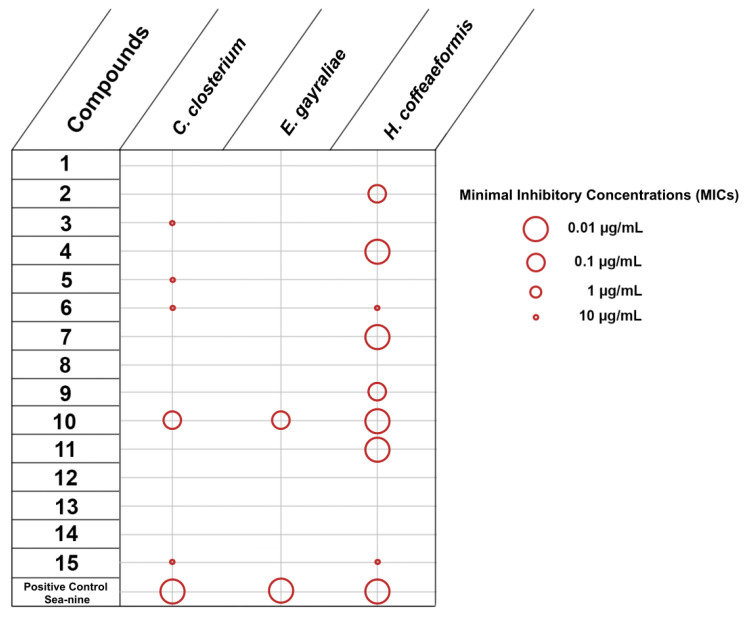
Inhibition of adhesion of microalgae by compounds **1–15** (MICs in µg/mL).

The initial set of experiments was designed to evaluate the inhibition of adhesion of five marine bacteria, namely *Vibrio aestuarianus*, *Vibrio harveyi*, *Vibrio natriegiens*, *Pseudoalteromonas citrea* and *Roseobacter litoralis*, and three microalgae, namely *Cylindrotheca closterium*, *Exanthemachrysis gayraliae* and *Halamphora coffeaeformis*. The obtained results were expressed as Minimal Inhibitory Concentrations (MICs) (Figure 2 and Figure 3).

Concerning the evaluation of compounds **1**–**15** against marine bacteria, among the 15 tested compounds, only compound **11** did not show activity. All other compounds inhibited at least two marine bacteria, with one (**2**), three (**1**, **6**, **12**), five (**3**, **5**, **8**, **9**, **13**) and five (**4**, **7**, **10**, **14**, **15**) molecules inhibiting two, three, four and all five marine bacteria, respectively (Figure 2). Compounds **4**, **7**, **10**, **14** and **15**, which showed inhibition of adhesion against all five marine pathogens were active only at the highest concentration (10 µg/mL). In contrast, compounds **1**, **6** and **12**, which inhibited the adhesion of three biofoulers could be considered as the most effective since most of the MIC values observed were below 0.1 µg/mL. *R. litoralis*, which faced the least impact on its growth, appeared the most sensitive, with 13 molecules inhibiting its adhesion. It is interesting to note that SeaNine is less efficient towards adhesion inhibition than the most active compounds and was not active at all against *P. citrea* and *R. littoralis*.

Among the 15 compounds tested for the inhibition of adhesion of microalgae, only compound **10** showed activity against all three microalgae (Figure 3). Only 14 responses affecting the adhesion of microalgae (compounds exhibiting activity x tested microalgae) were observed, of which half (for **2**, **4**, **7**, **9**–**11**) were recorded with MIC values of 10 µg/mL. *E*. *gayraliae* was the least impacted microalga, as only compound **10** was active towards its adhesion. SeaNine was very effective towards the inhibition of adhesion of microalgae, but it is known that the mode of activity is through toxicity.

**Figure 4:**
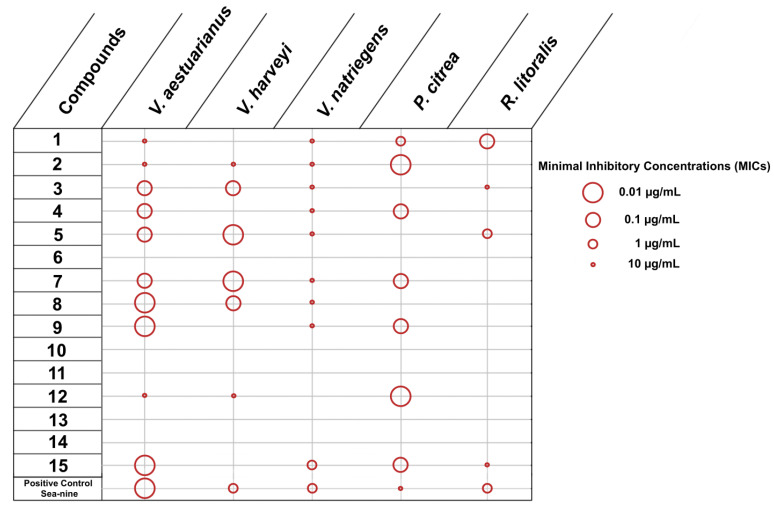
Inhibition of growth of bacteria by compounds **1**–**15** (MICs in µg/mL).

**Figure 5:**
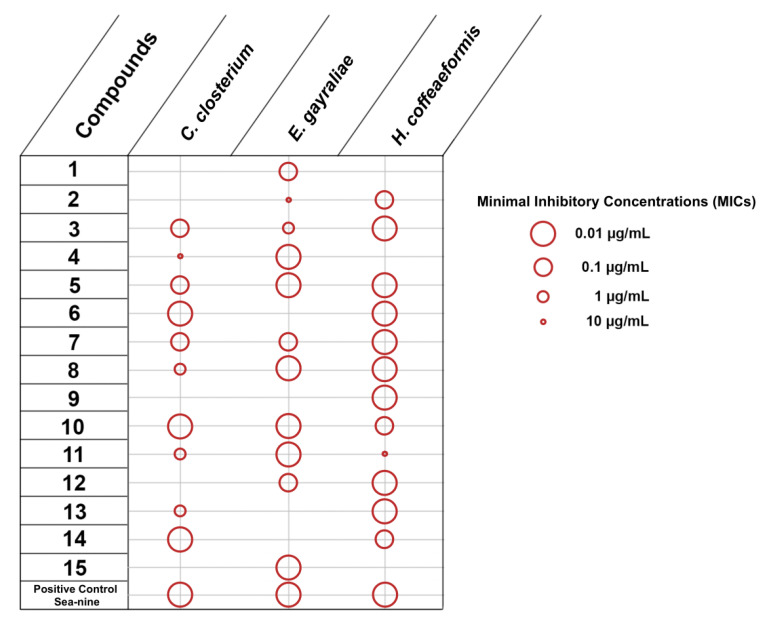
Inhibition of growth of microalgae by compounds **1**–**15** (MICs in µg/mL).

The same set of metabolites was evaluated for their activity against the growth of the same five marine bacteria and three microalgal species (Figure 4 and Figure 5).

According to the results (Figure 4), compounds **6**, **10**, **11**, **13** and **14** did not show any activity against the growth of any of the tested marine bacteria. However, compounds **1**–**3**, **5**, **7** and **15** showed activity against four out of the five marine bacteria tested, while compounds **4**, **8**, **9** and **12** inhibited three out of five marine bacteria. The most potent compounds with the lowest MIC values were compounds **5**, **7** and **15**. Among marine bacteria, *R*. *litoralis* was the most resistant, with its growth affected by compounds **3** and **15** at high concentrations (10 µg/mL) and compounds **1** and **5** at low concentrations (0.1 and 1 µg/mL, respectively). On the contrary, *V*. *aestuarianus* and *V*. *natriegens* were the most impacted, being susceptible to 10 and 9 compounds, respectively. Among them, *V*. *aestuarianus* appeared more sensitive than *V*. *natriegens*, since seven metabolites were active with MIC values ≤ 0.1 µg/mL. It is interesting to note that some compounds were more active than the positive control, which confirms the great potential of the algal metabolites for inhibition of bacterial growth.

Furthermore, all tested compounds inhibited the growth of at least one microalga with three (**1**, **9**, **15**), six (**2**, **4**, **6**, **12**–**14**) and six (**3**, **5**, **7**–**8**, **10**–**11**) molecules inhibiting one, two and all three microalgae, respectively (Figure 5). Among the 33 responses affecting growth of microalgae (compounds exhibiting activity x tested microalgae), 9 were recorded with MIC values of 0.1 µg/mL and 17 with MIC values of 0.01 µg/mL. Compounds **5** and **10** appeared to be the most effective in inhibiting the growth of the tested microalgae, closely followed by compound **4**. SeaNine toxicity towards microalgae was confirmed. The level of inhibition towards all the strains tested demonstrated that this compound is extremely deleterious to all photosynthetic organisms and that it is urgent to find alternative compounds with a more specific mode of action. 

#### 2.2.2. Inhibition of Bromoperoxidase and Phenoloxidase

Colonization, which is one of the most fundamental and precarious processes in the life history of benthic marine algae and invertebrates, regulates the distribution and abundance of organisms and therefore determines the community structure. 

When working on bioprospection, the choice of test is crucial. Colonization by invertebrates has been extensively studied, and the barnacle *A. amphitrite* has been used as a model species for many years. Nevertheless, in previous investigations, we have demonstrated that, in the context of studying the potential of marine natural products for invertebrate growth inhibition, most marine species investigated developed targeted compounds that were active against their primary colonizers. Thus, it is rare to discover compounds with broad-spectrum activity but without toxicity [48]. However, an exception was highlighted for invasive organisms that often have a very elaborate, broad-spectrum chemical defense arsenal. In order to evaluate the activity of compounds **1**–**15** for their anti-adhesion efficacy in marine invertebrates but without the constraint of carrying out biological tests on a whole panel of larvae, we decided to work on the inhibition of phenoloxidase, an enzyme conserved in many invertebrates [49].

Algae are understudied in the context of biofouling despite the fact that they are very successful in settling on a wide range of artificial substrata, such as oil and gas platforms, pilings, wrecks, mariculture facilities (nets and cages), ships, buoys and pontoons, and can cause significant damage, e.g., corrosion, decrease of the ships speed, technical problems in aquaculture systems, fishnets and power plant cooling systems [50]. Over the last decades, the occurrence and intensity of algal blooms has increased significantly due to anthropogenic activities and global climate changes. The phenomenon of global warming could deeply influence the evolution of fouling communities; a potential impact would be the reduction of calciferous species (e.g., barnacles, oysters) in favor of macroalgae [51]. The pressure of macroalgal fouling may be very high in the future. All these facts corroborate the idea that the search for inhibitors of algal fixation and adhesion should be a priority in order to be proactive regarding environmental changes. Nowadays, the main difficulty regarding antifouling assays using macroalgae is that they are highly time-consuming, season-dependent and species-dependent. In order to skip these major constraints, we have developed a new approach based on the use of oxidases (enzymes) which is one of the opportunistic strategies developed by numerous marine organisms, including macroalgae, for adhesion [1]. Testing compounds for their inhibitory activities towards oxidase-activated mechanisms represents a fast, reliable and targeted anti-algal assay [50].

Algal bioadhesives are constituted of a complex mixture of various organic compounds, including proteins, carbohydrates, glycoproteins, polyhydroxyphenols and metal ions, mutually interacting through cross-linked bonds as well as electrostatic forces and metal ion bridge complexes [52,53,54]. A model of oxidative cross-linking of secreted phenolics mediated via the catalysis of a bromoperoxidase (BPO) was highlighted in adherent spores of algae [55]. Hardening with alginate and/or calcium is essential for high cohesive strength as shown in shear-lap tests that measured the adhesion strength of this assemblage [52]. These data implicate BPO to be the key reagent in controlling the cross-linking of phenolic polymers for the assembly of algal adhesives [56]. This is why we decided to use BPO inhibition as a probe for anti-adhesion assay.

In invertebrates, such as mussels, oysters and barnacles, substrate attachment is established through adhesives whose formation is catalyzed by a copper-depending phenoloxidase (PO), which oxidizes phenolic residues, such as tyrosine, to catechols, such as 3,4-dihydroxy-l-phenylalanin (l-DOPA) [57]. By studying the enzymatic kinetics of PO, a specific antifouling test has been developed [57].

**Figure 6:**
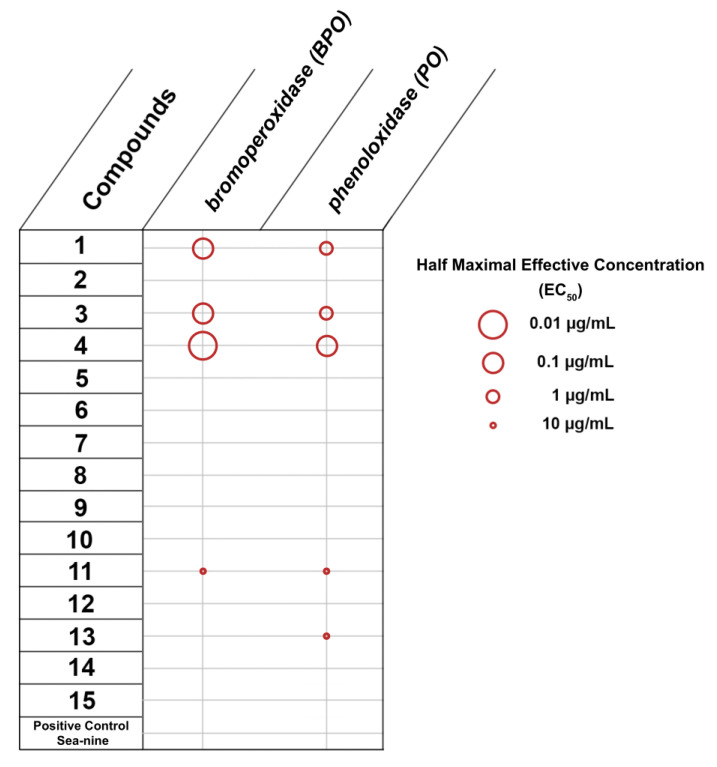
Inhibition of the activity of the bromoperoxidase and phenoloxidase by compounds **1**–**15**.

Compounds **1**–**15** were evaluated for their ability to inhibit bromoperoxidase (BPO) and phenoloxidase (PO). SeaNine was not active at all towards these enzymes (this is due to the fact that its mode of action is based on toxicity rather than on specificity). According to the obtained results, expressed as EC_50_ values (Figure 6) towards BPO and PO, compounds **2**, **4**, **5** and **12** were active in inhibiting both enzymes, with the highest activity observed for compound **5**, closely followed by compounds **2** and **4**. Compound **14** was active only towards PO. These results are very promising as macroalgae and invertebrates are very difficult to inhibit specifically and without toxicity. In addition, these tests have the advantage of targeting the adhesion process of many species of algae and invertebrates, and therefore the active compounds offer protection against a wide range of colonizing species using an oxidase-mediated process for adhesion; this is a great advantage because very often when an alga is inhibited, it is replaced by another species. 

#### 2.2.3. Toxicity Evaluation of the Compounds

At the concentrations tested, none of the compounds was toxic towards oyster larvae. For all compounds, LC_50_ values were above 100 µg/mL. These results are very promising and demonstrate the great potential of *S. coronopifolius* as a producer of non-toxic compounds active for the regulation of epibiosis.

### 2.3. Overall Antifouling Potential

Bromosphaerol (**1**) is an active molecule that has already been described as potential cancer stem-cell-targeting and antifouling agent [32]. In this study, thirteen bromosphaerol-related compounds (**2**–**14**) and the major sesquiterpene isolated from *S. coronopifolius* (**15**) were evaluated through five specific antifouling tests and demonstrated several interesting activities. Of the tested conditions, 70% and 69% resulted in positive hits against bacterial adhesion and microalgal growth, respectively. Less than 50% of tested conditions exhibited antifouling activity against bacterial growth, and less than 30% concerning algal adhesion and BPO/PO. These levels of inhibition are very interesting in terms of chemical ecology: indeed, the organisms that are the most inhibited correspond to the microbial colonizers, which are the most abundant in the marine environment and that pose constant threats to algae. In the case of *S. coronopifolius*, we can see that a very efficient arsenal of compounds is produced to inhibit biofilm formation. Fewer compounds were active against oxidase, but the level of activity recorded was very high (low EC_50_ values); this can reflect an induced type of defense, when targeted compounds are produced with a very specific mode of action (rather than acting by toxicity mechanisms, such as for growth inhibition). Among the tested metabolites, the most potent were compounds **3**–**5**, **7**, **10** and **15,** since they showed antifouling activity in more than 65% of the performed assays at different concentrations.

In 45% of the tests, compounds were proven inactive at the concentrations tested (up to 10 µg/mL). In 26% of the tests, compounds were active at 10 µg/mL, while in 4% they were active at 1.0 µg/mL. More importantly, in 12% and 13% of the bioassays, the compounds tested were active at 0.1 and 0.01 µg/mL, respectively. The compounds showing the most potent activity towards bacteria, microalgae and enzymes associated with macroorganisms at low concentrations were compounds **4**, **7**, **10** and **12,** and they appeared to be promising candidates for future studies, as performed in Alves et al. (2020) [58].

As far as diterpenes **1**–**14** are concerned, they share relatively similar oxygenated carbocycles decorated with at least one Br atom that is possibly important for the exhibition of activity on epibiotic organisms. Nonetheless, it is evident that the presence of the bromomethylene moiety enhances the compounds‘ activity, as evidenced by the significantly lower activity values exhibited by **13** and **14**. 

## 3. Materials and Methods

### 3.1. General Experimental Procedures

1D and 2D NMR spectra were recorded on a Bruker DRX 400 (Bruker BioSpin GmbH, Rheinstetten, Germany) spectrometer, using standard Bruker pulse sequences. Chemical shifts internally referenced to residual solvent signals are given on a *δ* (ppm) scale. Column chromatography separations were performed with Kieselgel Si 60 (Merck, Darmstadt, Germany). HPLC separations were conducted on a CECIL 1100 Series liquid chromatography pump (Cecil Instruments Ltd., Cambridge, UK) equipped with a GBC LC-1240 refractive index detector (GBC Scientific Equipment, Braeside, VIC, Australia), using a Kromasil 100-7-C_18_ (250 × 10 mm, Akzonobel, Eka Chemicals AB, Separation Products, Bohus, Sweden) for reversed-phase HPLC or a 250 mm × 10 mm i.d. Kromasil 100-10-SIL (Akzonobel, Eka Chemicals AB, Separation Products, Bohus, Sweden) for normal-phase HPLC. TLC was performed on Kieselgel 60 F_254_ (0.2 mm) precoated aluminum or glass plates (Merck, Darmstadt, Germany), and spots were visualized after spraying with H_2_SO_4_ in MeOH (20% *v*/*v*) reagent and heating at 100 °C for 1 min.

### 3.2. Biological Material

Specimens of *S. coronopifolius* were collected by scuba diving in Agios Giannis bay off Parga in Greece, at a depth of 4–20 m in July 2018. A voucher specimen of the alga has been deposited at the Herbarium of Section of Pharmacognosy and Chemistry of Natural Products, Department of Pharmacy, National and Kapodistrian University of Athens (ATPH/MP0601).

### 3.3. Extraction and Isolation

Algal specimens of *S. coronopifolius* were initially freeze-dried and then exhaustively extracted with mixtures of CH_2_Cl_2_/MeOH (3:1) at room temperature. The combined extracts were concentrated to give a dark green residue, which was later subjected to VCC on silica gel, using cHex with increasing amounts of EtOAc and finally MeOH as mobile phase to afford 12 fractions (1–12). Fraction 6 (50% EtOAc in cHex) was fractionated by gravity column chromatography, using a 5% step gradient of cHex/EtOAc. Fraction 6j (30% EtOAc in cHex) was further separated by normal-phase HPLC using cHex/EtOAc (82:18) as the mobile phase to afford compounds **1**, **4**, **9** and **15**. The CH_3_CN soluble part of fraction 7 (60% EtOAc in cHex) was subjected to reversed-phase HPLC, using CH_3_CN 100% as mobile phase and further purified by reversed-phase HPLC with MeCN/H_2_O (90:10) to yield compounds **2**, **5**–**7** and **10**–**13**. A final purification by normal-phase HPLC with cHex/CHCl_3_ (45:55) led to the isolation of compounds **3**, **8** and **14**.

### 3.4. Antifouling Evaluation Assays

Bioassays were conducted by employing key marine biofoulers: bacteria, microalgae, macroalgae and invertebrates. For all bioassays, compounds were assessed at concentrations of 0.01, 0.1, 1 and 10 µg/mL and on two batches of microorganisms and enzymes. For all bioassays, as a relevant positive control, data for the commercial antifouling booster Sea-nine™ are included (tested at concentrations from 0.01 to 10 µg/mL) [59].

These bioassays were performed to highlight the potency of the 15 compounds to inhibit the adhesion or growth of microorganisms and activity of enzymes of macroalgae and invertebrates involved in adhesion (bromoperoxidase and phenoloxidase, respectively).

Five marine bacteria known as model biofoulers and/or as marine pathogens were selected for this study: *Vibrio aesturianus* (DSM 19606), *Vibrio harveyi* (DSM 19623), *Vibrio natriegens* (DSM 759), *Pseudoalteromonas citrea* (DSM 8771) and *Roseobacter litoralis* (DSM 6996). Compounds were dissolved in methanol as a carrier solvent and, after evaporation, were incubated at the concentration of 0.01, 0.1, 1.0 and 10 µg/mL with bacteria (2 × 10^8^ cells/mL) in 96-well plates (MERCK) in 100 µL of MB medium (Marine Broth, Difco 2216) at 25 °C for 24 h. The measurement of growth was carried out by reading the optical density at 630 nm [33]. Results are expressed as the minimal inhibitory concentration (MIC) values. 

For the anti-adhesion test, wells were emptied and rinsed with 100 µL of sterile seawater to remove the non-attached cells. Wells were air-dried at room temperature and then the biofilm was stained with 100 µL of 0.1% of crystal violet for 30 min. Then, the previous steps were repeated, and the dried biofilms were resuspended and homogenized in 100 µL ethanol 80%. The control of adhesion was carried out by reading the optical density at 595 nm [60]. Results are expressed as MIC values.

Three microalgae characterized as the most common colonizers and known to be highly involved in biocorrosion processes were selected: *Cylindrotheca closterium* (AC 170), *Exanthemachrysis gayraliae* (AC 15) and *Halamphora coffeaeformis* (AC 713). Compounds were dissolved in methanol as a carrier solvent and after evaporation were incubated at the concentration of 0.01, 0.1, 1.0 and 10 µg/mL with algae in 96-well plates (MERCK) in 100 µL of f/2 medium at 20 °C during 5 days in constant light (incident irradiance: 140 µmol/m^2^/s). The microalgal concentration was estimated by correlation with chlorophyll a (Chl a) concentration [61]. The control of adhesion was carried out by reading the fluorescence density at 485 nm (excitation) and 645 nm (emission). Results are expressed as MIC values [60].

To evaluate the cell’s adhesion inhibition, the same conditions were used as stated above. After 5 days of incubation, 96 well plates were emptied. One hundred microliters of 100% methanol was added to liberated Chl *a*. The control of Chl *a* concentration (growth) was carried out by reading the fluorescence density at 485 nm (excitation) and 645 nm (emission). Results are expressed as MIC values [60].

In order to evaluate the anti-adhesion activity against macroalgae and invertebrates, oxidase enzymes were used as a probe. Thus, assays were performed using bromoperoxidase, an enzyme involved in adhesive-production by macroalgae, and phenoloxidase, an enzyme involved in adhesive production by mussels, barnacles and oysters.

Bromoperoxidase (BPO) inhibition assay: BPO activity, as a probe for macroalgae antifouling adhesion assay, was measured spectrophotometrically. The pure enzyme was purchased from Sigma (CAS no. 69279-19-2). For the enzymatic activity evaluation, an aliquot of the pure enzyme (corresponding to 0.1 unit/mL) was added to 0.375 mL of KBr 0.1 M (substrate of BPO) in 1.5 mL plastic UV-cuvettes, and production of bromophenol blue was monitored at 278 nm for 2 h. The results are presented as the concentration of compounds that reduces enzyme velocity by 50% (IC_50_ values). IC_50_ values were calculated using MINITAB.

Phenoloxidase (PO) inhibition assay: PO activity, as a probe for invertebrate antifouling adhesion assay, was measured spectrophotometrically as described earlier [62]. The pure enzyme (EC1.14.18.1) was incubated at 25 °C with 10 mM L-DOPA in a 50 mM phosphate buffer of pH 6.8. PO activity was determined by monitoring the increase in absorbance at 475 nm. One unit of enzyme activity was defined as the amount of enzyme that catalyzes the formation of 1 μmol dopachrome per minute under the described experimental conditions. The tested compounds were added at concentrations of 0.01 up to 10 μg/mL. Aliquots of the pure enzyme were incubated for 2 h in the presence of the compounds, and then the enzyme activity was recorded with L-DOPA (10 mM) as substrate. The results are presented as the concentration of compounds that reduces enzyme velocity by 50% (IC_50_ values). IC_50_ values were calculated using MINITAB.

### 3.5. Toxicity Assessment

Toxicity tests were conducted as previously described [63]. In order to determine the toxicity of the compounds on the development of larvae of oysters, *Crassostrea gigas* larvae were obtained from Argenton Marine Station (Lemar laboratory, University of Brest) and were incubated with a gradient of compounds’ concentrations (0.01, 0.1, 1.0, 10, 25, 50 and 100 µg/mL). Higher concentrations than those used for the antifouling assays were used in order to mimic a worst-case exposure scenario. Six replicates and two batches of larvae were used in order to reduce the effect of the natural variability between individuals. A population control comprised larvae reared in seawater. After 24 h at 20 °C, larvae reached the D stage (confirmed by microscopic observation). Larval development was then stopped by addition of 100 mL of formol to the culture medium. LC_50_ values were assessed using a log-logistic model (Kooijman, 1981). The model was fitted to the experimental data with the help of a non-linear regression program (SGPLUS, Manugistics, Incorporated, Rockville, MD, USA).

## 4. Conclusions

Among the 15 tested metabolites (**1**–**15**), compounds **3**, **5** and **15** were active against a wide range of biofoulers. They showed at least one positive response towards the panel of organisms tested, whether on the biofoulers’ growth or adhesion or on enzymes; they displayed a broad-spectrum type of activity. More precisely, compounds **1**–**10**, **12**–**15** were active against bacterial adhesion and compounds **1**–**5**, **7**–**9**, **12** and **15** against bacterial growth. Among them, compounds **1**, **6** and **12** were the most active with activities at lower concentration against bacterial adhesion, as well as compounds **5**, **7** and **15** against bacterial growth. Then, compounds **2**–**7**, **9**–**11** and **15** were active against microalgal adhesion and all compounds against microalgal growth. Among them, compounds **10** was the most active against microalgal adhesion, and most of the compounds (except for compound **2**, **4**, **11**) appeared active at concentration under 0.1 µg/mL against microalgal growth. Finally, compounds **1**, **3, 4**, **11** and **13** were active against BPO and/or PO. Among them, compound **4** appeared as the most potent with EC_50_ value < 0.1 µg/mL. Such metabolites have the potential to be used as booster compounds in antifouling formulations. 

None of the compounds tested were inactive, which confirmed the significant antifoulant potential of bromosphaerol (**1**) and compounds **2**–**15** isolated from *S. coronopifolius* [32,41]. These observations corroborate the fact that marine organisms use a combination of antifouling defenses, some molecules with a broad range of activities and some others displaying very specific modes of action. Moreover, it also corroborates the fact that diterpenes produced by algae are highly bioactive and associated with defense mechanisms [64,65,66]. Depending on the concentrations of each of these compounds and their combination (induced defense vs. constitutive defense), *S. coronopifolius* is able to efficiently control epibiosis on its surface. Marine macroalgae are known to adapt their chemical defenses to environmental conditions. Thus, some defences are synthesized constitutively and others inductively in order to best respond to epibiosis pressure. This is of particular interest since *S. coronopifolius* is a cultivable species (unpublished results) and it will be possible to apply OSMAC-like (One Strain MAny Compounds) approaches to grow it under various stress conditions to improve the commercial exploitation of its natural products [25,26,27,67,68]. Such studies could lead to the identification of other natural bioactive compounds with biotechnological applications. 

Further analysis by monitoring the production of these secondary metabolites throughout the year would permit the elucidation of the roles of these compounds and under which conditions their production is more pronounced. As a postulate, compounds **4**, **7**, **10** and **12** might be used by the red alga *S*. *coronopifolius* for its clean surface maintenance year-long, and other compounds could be produced as a response to an epibiosis-related stress. Additionally, in future applications, transcriptomic approaches would help to understand the biosynthetic pathways implicated in the production of such bioactive molecules, and then studied mixtures of these molecules to evaluate their biotechnological potential.
